# Coherent Nonlinear Optical Response Spatial Self-Phase Modulation in MoSe_2_ Nano-Sheets

**DOI:** 10.1038/srep22072

**Published:** 2016-02-26

**Authors:** Wenhui Wang, Yanling Wu, Qiong Wu, Jiaojiao Hua, Jimin Zhao

**Affiliations:** 1 School of Science, Xi’an Jiaotong University, Xi’an 710049, China; 2 Beijing National Laboratory for Condensed Matter Physics and Institute of Physics, Chinese Academy of Sciences, Beijing 100190, China

## Abstract

Two-dimensional (2D) transition metal dichalcogenides (TMDs) are drawing increasing interest due to their relatively high carrier mobilities, valley pseudospins, and gapped electronic structures, which all indicate interesting nonlinear optical properties of these 2D materials. However, such nonlinear optical properties are so far less investigated and their correlation with the electronic structure of the material is rarely probed. In this work, we have systematically investigated the spatial self-phase modulation (SSPM) of MoSe_2_ flakes in a suspension form, which is a coherent third-order nonlinear optical effect. The nonlinear susceptibility *χ*^(3)^ and its wavelength-dependence are measured, yielding a value of 1.1 × 10^−9^ e.s.u. (SI: 1.53 × 10^−17^ m^2^/V^2^) at 532 nm laser excitation for effective one-layer MoSe_2_.

The discovery of graphene has aroused tremendous interest in two-dimensional (2D) materials. Layered transition metal dichalcogenides (TMDs) are drawing increasing interest owing to their high carrier mobilities^1^,^2^,^3^,^4^ and semiconducting properties. For TMDs such as MoS_2_, MoSe_2_ and WTe_2_, a dramatic change occurs from an indirect bandgap in the bulk to a direct bandgap in the monolayer limit^5^. Moreover, these layered materials exhibit metallic, semiconducting or superconducting properties, depending on the coordination and oxidation states of the metal atoms. In addition, large effects of excitons^6^, strong photoluminescence^5^,^7^,^8^ and fully optical control of charge carriers in the valleys^6^,^9^,^10^ endow them with a wide range of applications in optoelectronics and valleytronics^7^,^11^,^12^,^13^,^14^,^15^. Besides the aforementioned electronic and linear optical properties, nonlinear optical properties of these novel quantum materials^16^,^17^,^18^,^19^,^20^,^21^,^22^,^23^ have also been investigated, which can enrich the comprehensive understanding of the behavior of carriers in 2D materials and extend solid basis for the potential optoelectronic applications^24^,^25^,^26^,^27^,^28^,^29^,^30^,^31^. Significantly, it has been demonstrated that SSPM provides a way to induce non-local ac electron coherence in the TMDs^16^ and measure their nonlinear optical susceptibilities. Here we carry out a parallel investigation on another TMD, MoSe_2_, to measure its wavelength-dependent *χ*^(3)^.

Investigating the nonlinear optical properties of these novel materials is quite essential since, if the optical properties of these newly discovered quantum materials are superb, it will for the first time make it possible to integrate electronic and optoelectronic devices in one material system, which is so far challenging to realize in the indirect-gap silicon and no-gap graphene systems. Among such optical properties, nonlinear optical properties are even more critical, because, besides lasing itself, most of the photonics applications are based on nonlinear optical properties^32^,^33^,^34^,^35^,^36^. For example, optical switches, which are as important to photonics as transistors to electronics, are all realized using various nonlinear optical effects (*e.g.*, four-wave mixing^37^, three-wave mixing^38^, SSPM^16^, *etc*.)—the switching itself can only be a nonlinear process. In terms of nonlinear optical processes, the second harmonic generation only occurs in materials without inversion symmetry (*i.e.*, limited materials, most of which cannot be integrated)^39^, and the forth-order and higher-order nonlinear optical effects are usually much weaker than the third-order responses. Therefore it is essential to obtain the knowledge about the third-order nonlinearity *χ*^(3)^^16^,^17^,^24^,^40^,^41^,^42^,^43^. Knowing its magnitude and dependence on the working wavelength is very useful in selecting the materials and designing an optoelectronic device.

Spatial self-phase modulation, which was initially investigated in nematic liquid crystal in 1981^44^, is a purely coherent nonlinear optical effect that is characterized by an intensity-dependent refractive index *n* = *n*_0_ + *n*_2_*I*, where *n*_0_ and *n*_2_ are linear and nonlinear refractive indices and *I* is the laser intensity. The effect is also known as optical Kerr effect, which can induce self-focusing in the crystal or other type of materials when the effect is strong enough. The SSPM is a third-order nonlinear optical process (parallel to that of third harmonic generation and four-wave mixing), which is characterized by an additional transverse wavevector developed due to the transverse gradient in refractive index. When passing through the sample, the optical phase of incident beam depends on the transverse light intensity distribution, resulting in a conical out-going diffraction and interference in the far field (as the concentric fringes). As a result, the outgoing beams do not propagate along the incidence direction anymore. The rings are prominently different from the well-known Newton’s ring (a linear optics phenomenon) in that the ring number *N* in SSPM is finite and linearly dependent on the laser fluence. In addition, the outermost ring is the thickest among all the concentric fringes. In SSPM investigations, *N* directly reflects the optical phase (*i.e.*, the exact nonlinear effect as reflected by *n*_2_ and *χ*^(3)^) and is correlated to the electronic phase and coherence of the material^16^. Thus it is a more essential and intrinsic observable than the ring diameter *D*, because *D* can also be modified by the linear refractive index *n*_0_.

Unlike other nonlinear optical effects, the effect of SSPM can be observed conveniently by using both continuous wave (cw) and ultrafast lasers with only milliwatts laser powers. Furthermore, the nonlinear refractive index *n*_2_, hence the third-order nonlinear susceptibility *χ*^(3)^, can be directly and quantitatively obtained through a convenient way^16^,^17^. Therefore, this experimental method has been developed to be a general way for measuring the *χ*^(3)^ of 2D layered quantum materials^16^,^17^. It applies to the broad spectrum-regime including the violet and ultra-violet regime, where third harmonic generation and four-wave mixing are ineffective. It can also be applied to the situation when large single crystal is hard to achieve. Significantly, since a wind-chime model^16^ has been developed to account for the emergence of ac electron coherence in the materials, this method has endowed the capability of comparing and correlating to the electronic band structure of a specific layered quantum material.

In this work, we directly measured the wavelength-dependent coherent nonlinear optical response SSPM of nanoscale MoSe_2_ sheets, and obtained their *χ*^(3)^(*ω*) values. Comparison with absorption has been carried out, further demonstrating SSPM as a useful method in probing the electronic band structure of layered materials.

## Results and Discussion

**Figure 1:**
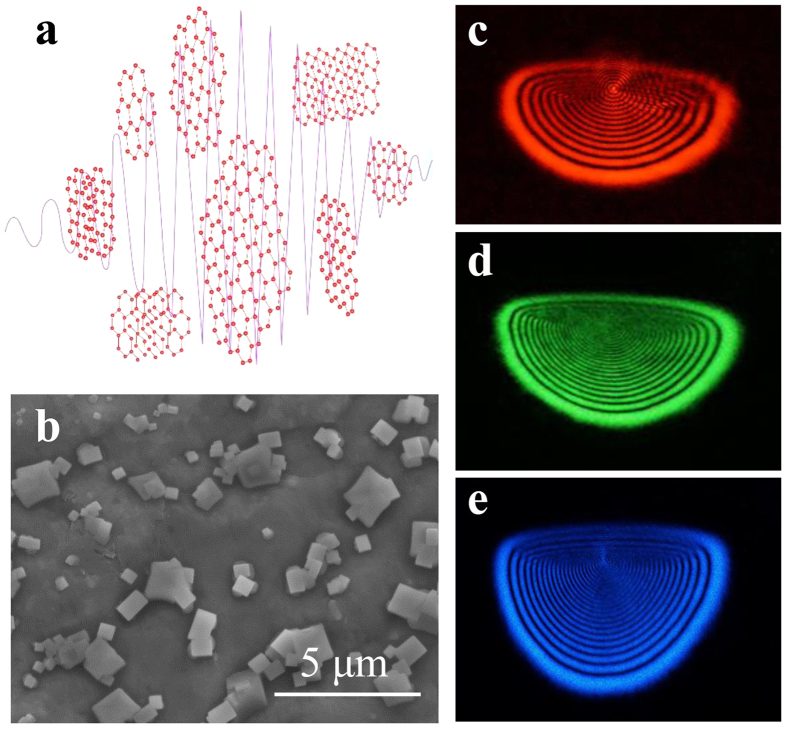
(**a**) Schematic illustration of the interaction between incident light and the MoSe_2_ flakes. (**b**) A SEM image of the MoSe_2_ flakes. (**c**–**e**) Typical SSPM patterns generated from the MoSe_2_ suspension under laser excitations with wavelengths of 735 nm, 532 nm, and 473 nm, respectively.

Linearly polarized cw laser beams with various wavelengths have been employed to excite the MoSe_2_ suspension (*Methods and Materials*). The schematics of our experimental setup is shown in Fig. S1 (*see supporting information*). The interaction between the incident light and the MoSe_2_ flakes is schematically illustrated in Fig. 1a, where the red honeycomb lattices represent the MoSe_2_ flakes. As a result of the interaction, these flakes are rotated and aligned to be along the light polarization direction. A typical scanning electron microscope (SEM) image of the MoSe_2_ flakes is shown in Fig. 1b, where the dimension of most of the flakes is about 0.3~2 μm. As shown, most of the MoSe_2_ flakes in our experiment are multi-layered bulk materials. In Fig. 1(c–e) we show typical SSPM diffraction ring patterns for the 735 nm, 532 nm and 473 nm excitations, respectively, which are concentric rings resulted from the conical out-coming beams. Note that the outermost ring is thickest and has the strongest intensity, with those of others decrease towards the center. The calculation result of the radial intensity distribution is shown in Fig. S2 (*see supporting information*). It can be seen that the calculation result compares well with the experimental observation shown in Fig. 1d. For each laser wavelength, the ring pattern is clear and uniform. The fringes have sharp edges, and their thicknesses become narrower when the laser beam covers more number of the flakes^16^. To make sure that the concentric diffraction rings are not resulted from the solution itself, we have carried out a control experiment where the pure suspension without any MoSe_2_ flakes was used. It can be seen in Fig. S3 (*see supporting information*) that no diffraction rings can be observed on the white screen at the far field when a 532 nm laser beam was introduced, even though the laser power is increased to our laser’s maximum output 355 mW.

**Figure 2:**
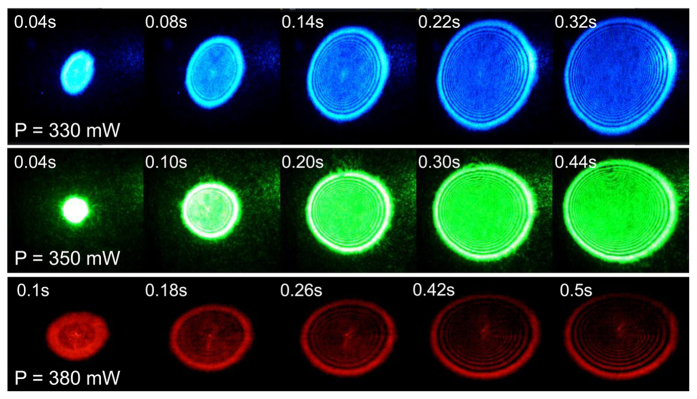
Snapshots of the SSPM ring pattern formation process under laser excitations with wavelengths of 735 nm, 532 nm, and 473 nm, respectively. The whole formation process takes 0.3~0.5 s.

To focus on the pattern formation process, we show the snapshots of the emergence of the SSPM rings in Fig. 2. It can be seen that the number of rings increases steadily with time until the maximum is reached after around 0.3~0.5 s. This temporal evolution of the pattern formation is well explained by our recently proposed wind-chime model^16^. Taking the model, the 0.4 s time duration is the time interval needed for the rotation and alignment of the flakes by the incident light—the emergence of the electron coherence and its maximization. The electrons at excited state are forced to oscillate at the excitation optical frequency, which further emit light to form the diffraction rings at the far field. Without the presence of a laser beam, each MoSe_2_ flake suspended in the solution can be treated as an independent domain, whose orientation is arbitrary. Thus, the phases of the electronic wave functions^16^ of the photo-induced charge carriers or holes or excitons in each domain are completely irrelevant. However, when a polarized laser beam is applied, each flake will get aligned in a way that they have an axis parallel to the light polarization. This aligning process is achieved due to the energy relaxation. When the MoSe_2_ flakes are fully aligned, the photon-induced carriers in each domain experience reduced boundary scattering, leading to less stray lights in the diffraction. Therefore, this SSPM process becomes a nearly purely coherent nonlinear optical process. Other than this purely coherent factor, the reduction of the refractive index heterogeneity also leads to enhanced coherence, because the light absorption, carrier mobility and effective mass are different along *x*, *y* and *z* axes.

The subsequent deformation (not shown in Fig. 2, but can be seen in Fig. 1c–e) can be attributed to a thermal effect, where the density of the solution becomes asymmetric under laser heating and gravitation^17^,^45^— the linear index *n*_0_ is modified to be non-uniform along the vertical direction. During this latter process, *n*_2_ and *χ*^(3)^ do not change since the number of rings *N* remains unchanged. It is worth noting that there is so far no direct experimental observation of a thermal gradient Δ*T* for the initial ring formation process (Fig. 2), although Δ*T* is frequently assumed as the starting point in many theoretical simulations. Note that this is quite different from the situation for the latter ring deformation process (Fig. 1c–e). In our experiment, the rings all develop to full circle till the maximum number of rings is reached. No pattern deformation occurs during the increase of the ring number (Fig. 2). Since the initial pattern formation process is clearly seperated from the next (following) pattern deformation process, thermal effect does not contribute to the initial pattern formation process (Fig. 2). Thermal effect only contributes to the latter (following) pattern deformation process. Most of the laser heat is dissipated (in the deformation process) through transfering to the solution and ultimately gone by evaporization.

**Figure 3:**
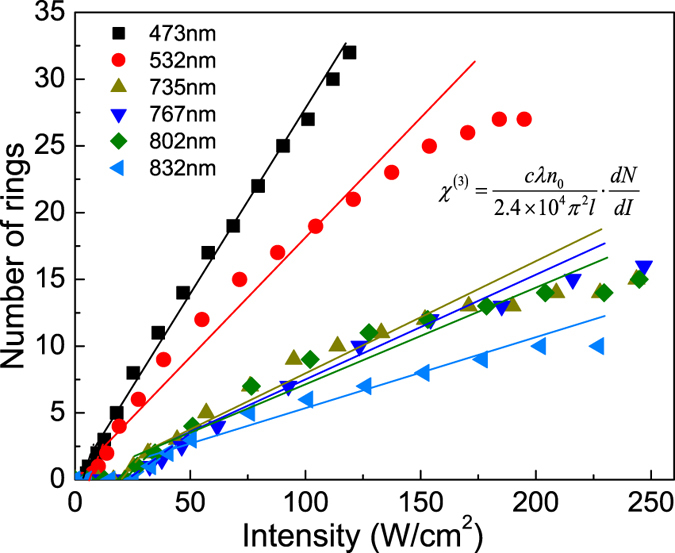
Dependence of the ring number *N* on incident laser intensity *I* for different wavelengths. The solid lines are guides to the eye for the overall linear relation and the corresponding slopes directly yield the third-order nonlinear optical susceptibility *χ*^(3)^ for different wavelengths.

The intensity dependence of the ring number *N* for different wavelengths are shown in Fig. 3. It can be seen that *N* increases nearly linearly with the laser beam intensity *I* and the slops of the data vary with the laser wavelength (*i.e.*, the excitation photon energy). Consequently, the values of *n*_2_ can be directly obtained from the slopes in Fig. 3 with *n*_2_ = (*λ*/2*n*_0_*L*) (*N*/*I*), where *L* is the thickness of MoSe_2_ suspension. Taking *L* = 10 mm, *n*_0_ = 1.47, we acquire that *n*_2_ = 3.24 × 10^−10^ m^2^/W for a 532 nm laser beam. Based on the one-to-one correspondence, the magnitude of the third-order nonlinear susceptibility can be obtained according to the relation^16^,^17^ *n*_2_ = (12*π*^2^/*n*c)10^3^*χ*^(3)^, which yields a value of 1.8 × 10^−4^ (e.s.u.). The value of *χ*^(3)^ for effective one-layer MoSe_2_ can be estimated from *χ*^(3)^_total_ = *M*^2^ *χ*^(3)^_one-layer_, where *M* is the effective number of MoSe_2_ layer that the laser beam traverses through. By estimating *M* to be around 400 in our experiment [*Methods and Materials*], we obtain that the value of *χ*^(3)^ for effective one-layer MoSe_2_ is about 1.1 × 10^−9^ e.s.u.

**Figure 4:**
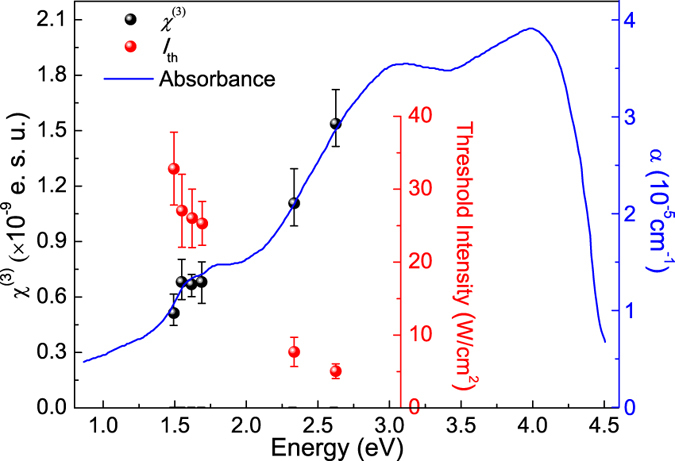
Comprehensive illustration of the *χ*^(3)^ (black dots) and intensity threshold *I*_th_ (red dots) for the near-infrared to visible spectral range. The blue solid curve is adopted from ref. 34 and illustrates the absorption spectrum for a sputtered MoSe_2_ thin film. The well comparison between the *χ*^(3)^ data with the absorption curve shows their proportionality.

We further investigated the wavelength dependence of the SSPM effect. The *χ*^(3)^ and intensity thresholds at various wavelengths have been measured and shown in Fig. 4, respectively. It can be seen that *χ*^(3)^ increases steadily with decreasing wavelength. Significantly, its value is well proportional to the light absorption measured by J. Pouzet *et al.* for sputtered MoSe_2_ layers^46^. The correlation between *χ*^(3)^ and absorption demonstrates that *χ*^(3)^ is determined by the band structure. In the photo-generation of free carriers or excitons, the quantum transition probability is proportional to the occupation number of the valance and conduction bands. The integration of such transition probabilities in the momentum space yields a value that is proportion to the *χ*^(3)^ value we measured here. Therefore, our current experiment further demonstrates that SSPM can be an effective method to probe the integrated information of the electronic band structure of a layered material. It can be used to experimentally verify whether the theoretical calculation of the band structure is correct—as absorption spectroscopy can do. And the wavelength-dependence of *χ*^(3)^ is sensitive to different layered materials, which can certainly be useful in characterizing new materials in the future. Here, at relatively higher photon energies, some of the in-active transitions become accessible; thus the value of *χ*^(3)^ is higher for higher photon energies and the value of threshold is lower for higher photon energies, correspondingly (Fig. 4).

During the above process, the outermost ring thickness and the ring diameter also show linear dependences on *I*, ending by the frequently observed thermal effect causing a “half-ring”^17^,^45^. As discussed above, *D* can be directly affected by not only *n*_2_ but also *n*_0_, thus a less important observable. However, the linear dependence of *D* on *I* makes the SSPM effect practical for all-optical switching^16^, particularly with that a weak beam can control a strong beam with high contrast ratio^16^,^38^. The TMD materials can be integrated and tailored, and the switches can be cascaded. Thus our nonlinear optical investigation here further demonstrates the great potential for TMDs in ultrafast photonics applications.

In conclusion, we have unambiguously observed the nonlinear optical response SSPM in a suspension of MoSe_2_ flakes, yielding a value of 1.1 × 10^−9^ e.s.u. (*i.e.*, 1.53 × 10^−17^ m^2^/V^2^) for the nonlinear dielectric susceptibility *χ*^(3)^ for an effective one-layer at 532 nm excitation. The wavelength-dependence *χ*^(3)^ = *χ*^(3)^ (*ω*) was measured for MoSe_2_, which is proportional to that of the light absorption. This confirms that SSPM is a ubiquitous property of a layered quantum material, which can be well described by the wind-chime model we previously developed^16^. Our investigation further demonstrates application potentials of MoSe_2_ and other TMDs in photonics, such as all-optical switching and optical limiting.

## Methods and Materials

The sample preparation is similar to that in Ref. [16] for MoS_2_. Micrometer-sized MoSe_2_ powder (LSKYD, http://www.kydmaterials.com/en/index.php) was added into a N-methyl-2-pyrrolidinone (NMP) solvent, followed by 30 minutes of ultrasonication. The MoSe_2_ flakes are uniformly distributed and suspended in the solution without observable aggregation. The density (*C*) of MoSe_2_ is 7.1 × 10^−4^ mol/L. In the experiment, linearly polarized cw laser beams with various wavelengths (473 nm, 532 nm 735 nm, 767 nm, 802 nm and 832 nm) were focused onto the MoSe_2_ suspension using a lens of 200 mm focal length. The illuminated area in the suspension is estimated to be 0.182 mm^2^ with a 1/e^2^ intensity radius of 0.24 mm. The light at the out-coming surface is diffracted off the initial propagation direction. At propagating to the far field, they interfere with each other to form conical output beams, which produce sharp concentric fringes on the white screen.

The number of effective layers of the MoSe_2_ flakes that the laser beam passes through can be estimated as follows. The volume of the solution (*V*) is 4 × 10^−3^ L. The total number of (MoSe_2_) molecules in the solution is *C* × *V* × *N*_A_ (=1.71 × 10^18^), where *N*_A_ is the Avogadro’s number. Meanwhile, since the area of the cross-section of the cuvette is *S* (=1 cm × 4 cm), the total number of molecules in one effective layer is *S*/a^2^, where a (=3.299 Å) is the lattice constant of MoSe_2_. Thus, the number of effective layers can be obtained as *C* × *V* × *N*_A_/(*S*/a^2^) = 403 ≈ 400.

When we use SSPM to measure *χ*^(3)^ (*ω*), it is necessary to make sure the saturation due to sample concentration will not occur and the sample itself is very stable during the measurement. To do so, it is suggested to make sure that the sample concentration is relatively low such that there is no precipitation of flakes and it is as low as being able to measure the fringe numbers conveniently.

## Additional Information

**How to cite this article**: Wang, W. *et al.* Coherent Nonlinear Optical Response Spatial Self-Phase Modulation in MoSe_2_ Nano-Sheets. *Sci. Rep.* **6**, 22072; doi: 10.1038/srep22072 (2016).

## Supplementary Material

Supplementary Information
