# Supplementary material for: Coherent Nonlinear Optical Response Spatial Self-Phase Modulation in MoSe2 Nano-Sheets
Source: Sci Rep. 2016 Feb 26;6:22072. doi: 10.1038/srep22072 (PMC4768144; doi:10.1038/srep22072)
Supplement: Supplementary Information [file srep22072-s1.doc]

**­­­Coherent Nonlinear Optical Response Spatial Self-Phase Modulation in MoSe_2_ Nano-Sheets**

**Wenhui Wang^1^, Yanling Wu^2^, Qiong Wu^2^, Jiaojiao Hua^1^, Jimin Zhao^2^,***

*^1^ School of Science, Xi'an Jiaotong University, Xi'an 710049, China*

*^2^ Beijing National Laboratory for Condensed Matter Physics and Institute of Physics, Chinese Academy of Sciences, Beijing 100190, China*

** Corresponding author, Email:* [*jmzhao@iphy.ac.cn*](mailto:jmzhao@iphy.ac.cn)

By using the sample preparation procedure described in *Methods and Materials,* we carried out the SSPM experiment, and the experimental setup is schematic illustrated as below. A linearly polarized cw laser beam with certain wavelength is focused onto the suspension of MoSe_2_ flakes through a lens of 200 mm focal length. The conical out-coming light can form concentric diffraction rings in the screen at far field.


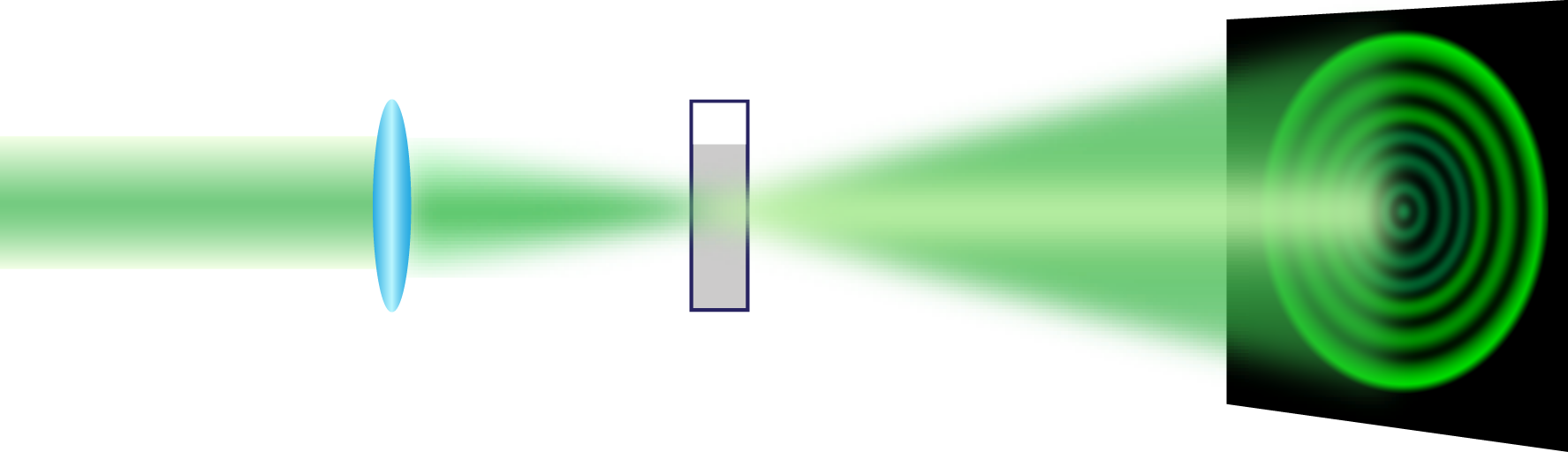


**Figure S1.** Schematics of the experimental setup.

The intensity distribution of the diffraction rings has been measured, and the result for 532 nm laser is shown in the following (Fig. S2a). We extracted the data along a diameter line (Fig. S2b). It can be seen that the outmost ring has the strongest intensity and the thickest width, which both decrease towards the center. The theoretical calculation result is also shown (Fig. S2c), which is in good agreement with our experimental results (Fig. S2a & S2b).


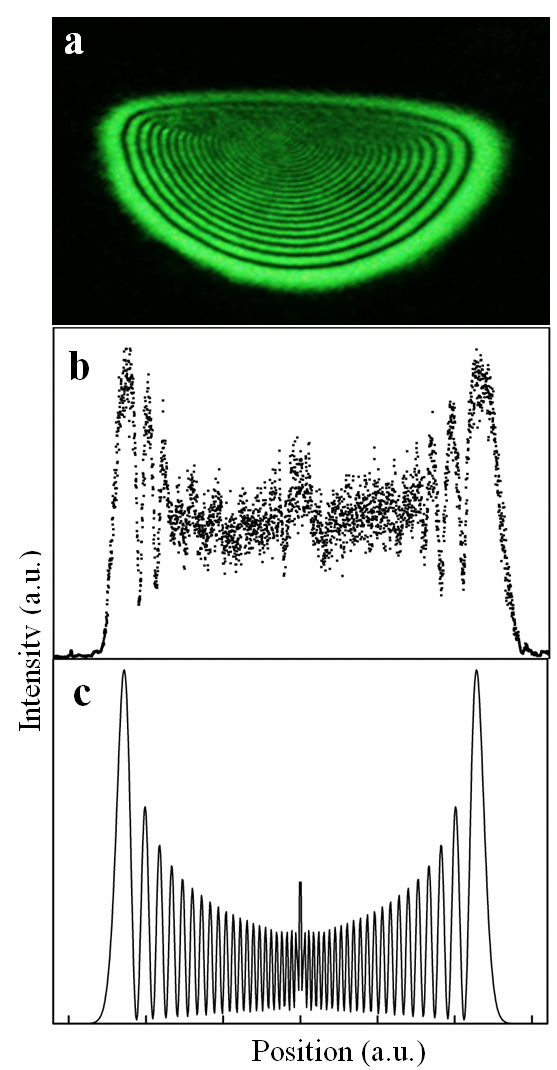


**Figure S2.** (a) Photograph of a typical SSPM ring pattern at 532 nm excitation. (b) Radial intensity distribution of the ring pattern shown in Fig. S2a. (c) Theoretical calculation result for the radial intensity distribution of the ring pattern shown in Fig. S2a. a.u. represents arbitrary units.

To confirm that the concentric rings at far field are resulted from the electron coherence in the MoSe_2_ flakes, we carried out a contrast experiment under the same experimental conditions except that pure solution without any MoSe_2_ flakes was investigated. A 532 nm laser beam was used. The result (Fig. S3) shows that no diffraction ring can be observed on the white screen during the whole process of increasing the laser power. The laser power was increased to our laser’s maximum output (355 mW), which far exceeds the threshold for observing a SSPM ring in the far field (14 mW).


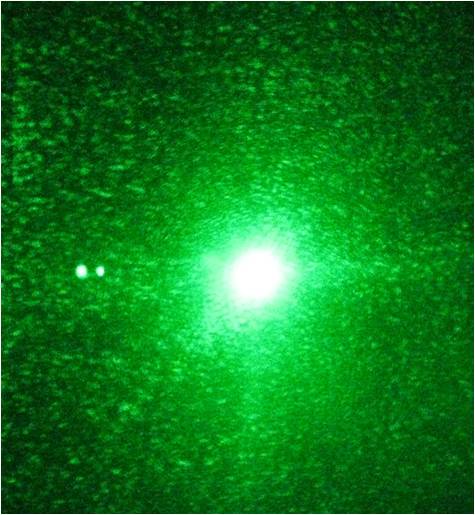


**Figure S3.**  Photograph taken for a pure NMP solvent without MoSe_2_ flakes. All the setup is identical for the SSPM experiment done for that with MoSe_2_ flakes. The laser wavelength is 532 nm and the laser power is 355 mW.
